# Salt concentration in substrate modulates the composition of bacterial and yeast microbiomes of *Drosophila melanogaster*

**DOI:** 10.20517/mrr.2023.56

**Published:** 2024-02-28

**Authors:** Ekaterina Yakovleva, Irina Danilova, Irina Maximova, Alexander Shabaev, Anastasia Dmitrieva, Andrey Belov, Alexandra Klyukina, Ksenia Perfilieva, Elizaveta Bonch-Osmolovskaya, Alexander Markov

**Affiliations:** ^1^Biological Faculty, Lomonosov Moscow State University, Moscow 119991, Russia.; ^2^Faculty of Soil Science, Lomonosov Moscow State University, Moscow 119991, Russia.; ^3^A.N. Bach Institute of Biochemistry, Research Center of Biotechnology, Russian Academy of Sciences, Moscow 119071, Russia.; ^4^Winogradsky Institute of Microbiology, Federal Research Center of Biotechnology, Russian Academy of Sciences, Moscow 117312, Russia.; ^5^Borisyak Paleontological Institute, Russian Academy of Sciences, Moscow 117997, Russia.

**Keywords:** Fruit fly, holobiont, adaptation, high salinity substrate, *Acetobacter*, lactobacteria, bacterial and yeast microbiota

## Abstract

**Aim:** Microbiomes influence the physiology and behavior of multicellular organisms and contribute to their adaptation to changing environmental conditions. However, yeast and bacterial microbiota have usually been studied separately; therefore, the interaction between bacterial and yeast communities in the gut of *Drosophila melanogaster* (*D. melanogaster*) is often overlooked. In this study, we investigate the correlation between bacterial and yeast communities in the gut of *D. melanogaster*.

**Methods:** We studied the shifts in the joint microbiome of *Drosophila melanogaster*, encompassing both yeasts and bacteria, during adaptation to substrate with varying salt concentrations (0%, 2%, 4%, and 7%) using plating for both yeasts and bacteria and NGS-sequencing of variable *16S rRNA* gene regions for bacteria.

**Results:** The microbiome of flies and their substrates was gradually altered at moderate NaCl concentrations (2% and 4% compared with the 0% control) and completely transformed at high salt concentrations (7%). The relative abundance of *Acetobacter*, potentially beneficial to *D. melanogaster*, decreased as NaCl concentration increased, whereas the relative abundance of the more halotolerant lactobacilli first increased, peaking at 4% NaCl, and then declined dramatically at 7%. At this salinity level, potentially pathogenic bacteria of the genera *Leuconostoc* and *Providencia* were dominant. The yeast microbiome of *D. melanogaster* also undergoes significant changes with an increase in salt concentration in the substrate. The total yeast abundance undergoes nonlinear changes: it is lowest at 0% salt concentration and highest at 2%-4%. At a 7% concentration, the yeast abundance in flies and their substrate is lower than at 2%-4% but significantly higher than at 0%.

**Conclusions:** The abundance and diversity of bacteria that are potentially beneficial to the flies decreased, while the proportion of potential pathogens, *Leuconostoc* and *Providencia*, increased with an increase in salt concentration in the substrate. In samples with a relatively high abundance and/or diversity of yeasts, the corresponding indicators for bacteria were often lowered, and *vice versa*. This may be due to the greater halotolerance of yeasts compared to bacteria and may also indicate antagonism between these groups of microorganisms.

## INTRODUCTION

*Drosophila melanogaster* (*D. melanogaster*) is a classical object used to study the microbiome and its role in the host’s life. The fly microbiome consists of two main components: yeasts and bacteria. Its composition is less diverse compared to mammals, which makes it easier to trace the influence of various external factors, making *D. melanogaster* a convenient model object for studying the relationship between the host and its microbiome^[1-7]^.

In addition, *D. melanogaster* is a convenient object for experimental studies of the organism’s adaptation to new conditions, including its response to a substrate with high NaCl content. High salinity serves as a convenient and frequently used factor for studying the adaptation process. The adaptation of *D. melanogaster* to a substrate with high salinity aligns with the objectives of evolutionary studies, as this factor is atypical for wild *Drosophila*. It not only represents a novel environment for them but also an unfavorable one. It was found that a substrate with a salt content exceeding 2% increased the mortality of *D. melanogaster* larvae or significantly delayed their development. At a concentration above 4%, the flies usually died within 4-5 days, accompanied by a sharp decrease in the number of eggs laid^[8,9]^. However, after several decades of generations living on a diet with a progressively increasing salinity, laboratory populations became adapted to the substrate with 6%-7% NaCl^[8,10]^ and even 7%-8%^[11,12]^.

According to previous studies, the bacterial microbiota of the fly gut is primarily dominated by representatives of one, two, or three taxa: the order *Lactobacillales* (phylum *Bacillota*) and the families *Enterobacteriaceae* and *Acetobacteraceae* (phylum *Pseudomonadota*). In laboratory-reared *D. melanogaster*, the bacterial component of the microbiome is generally less diverse than in wild populations and, in most cases, is represented by two physiologically distinct groups^[13]^. The first group includes obligately aerobic members of the *Acetobacter* genus, mainly *A. pomorum* and *A. pasteurianus*. The second group is comprised of aerotolerant bacteria of the family *Lactobacillaceae*, previously classified in the genus *Lactobacillus* but now reclassified^[14]^ into representatives of the genera *Lactiplantibacillus* (*L. plantarum*) and *Levilactobacillus* (*L. brevis*). The specific composition of the fly microbiota varies and is highly dependent on the environmental conditions, including their diet^[15]^.

It was previously believed that the intestinal microbiota of *D. melanogaster* is mainly transient, entering the gut with the substrate, and its development in the gut itself is minimal^[16]^. In contrast, recent research suggests that certain bacteria establish more complex and specific interactions with the fly gut, contributing to a stable gut bacterial microbiota and providing benefits to the host^[17-20]^. Notably, *L. plantarum* and some other bacterial symbionts stimulate larval growth^[13,21]^, increase adult longevity^[22]^, and improve food resource utilization efficiency^[1]^. *A. pasteurianus* produces thiamine required by flies^[23]^. *L. brevis* can influence the motor activity of flies by altering the sugar levels and indirectly regulating neuronal activity through the synthesis of the neurotransmitter octopamine^[24]^. Given these findings, it is logical to assume that bacteria may also influence the adaptation of flies to new conditions, including salty substrate, though the question has not yet been thoroughly studied.

Yeasts are another indispensable component of the microbiome of *D. melanogaster*, playing a crucial role in their life cycle. Yeast serves as an essential source of protein, along with other nutrients and micronutrients required for larval development^[25-32]^. Larvae reared on yeast-poor substrates exhibit reduced immunity, have a diminished ability to adapt to unfavorable conditions, and often fail to complete development^[27,29-31]^. On the other hand, yeast-poor diets can enhance cold tolerance^[33]^ and increase the longevity of *D. melanogaster*^[34,35]^. The yeast content in the substrate can have opposing effects on longevity, fecundity, and mating frequency of fly females and males^[36]^. In turn, flies influence the species composition of fungal communities inhabiting forage substrates, favoring the development of some yeast species and suppressing the growth of other yeasts and micromycetes^[37]^. Larvae tend to favor yeast species that promote faster development and increased adult body mass^[4,27]^. Volatile aromatic compounds released by yeasts attract fruit flies to fermented plant substrates, which, without yeast assistance, are not as appealing to flies. Combined with the ability of some yeasts to remain viable as they pass through the digestive tract of *D. melanogaster*, this enables yeasts to utilize fruit flies for their dispersal^[38-40]^. Previous studies have demonstrated that the abundance and species composition of yeasts associated with *D. melanogaster* may depend on the salt concentration in the substrate. Certain yeast species, such as *S. bacillaris*, may contribute to the adaptation of laboratory fly lines to salty substrates^[41-44]^. Evidently, the yeast community of flies adapts to the salty substrate alongside the host organism, reflecting the adaptability of the entire symbiotic complex. This study, along with our earlier works^[45]^, highlights the role of the microbiome in general and yeasts in particular in the adaptation of flies to salty substrate.

We did not find any studies investigating the transformation of the *D. melanogaster* microbiome during adaptation to changing environmental conditions. Furthermore, the yeast and bacterial components of the microbiome are usually studied separately, potentially leading to the oversight of significant interactions between them. In this study, we compared the bacterial and yeast components of the microbiome of *D. melanogaster* reared on substrates with different NaCl concentrations for seven years to identify trends in microbiome changes as flies adapt to increasing salinity. Parallel studies of yeast and bacterial communities of *D. melanogaster* aim to clarify the role of the microbiome in the host’s adaptation to changing environmental conditions and shed light on the relationships within a dynamically changing microbial community.

The primary objective of this study was to investigate the changes in the bacterial component of the fly microbiota during host adaptation to increasing salt concentration in the substrate. The secondary, yet crucial aim, was to elucidate the interaction between bacterial and yeast components of the fly microbiota.

## METHODS

### Samples selection and preparation

Adult *D. melanogaster* of outbred laboratory lines obtained from the wild individuals collected in southwest Moscow (Russia) in October 2014 were used. Until January 2015, the initial fly population was cultured on standard medium (inactivated baker’s yeast - 60 g, semolina - 35 g, sugar - 50 g, crushed raisins - 45 g, agar CAS 9002-18-0 - 8 g, propionic acid CAS 79-09-4 - 2 g per 1 liter of medium)^[46]^. Three control (0a, 0b, and 0c) and 8 “salt diet” lines were isolated then from this initial fly population to model the evolutionary process.

Control lines were reared on a standard medium without salt. Three lines labeled 2a, 2b, and 2c were reared on a standard medium supplemented with 2% NaCl from 2014 until the start of the study. Three lines labeled 4a, 4b, and 4c were reared on a standard medium with 4% NaCl from 2014 until the start of the study. Two lines labeled 7a and 7b were reared on a 4% NaCl medium until 2016, and then the salt concentration was increased by 0.5% every 2-3 months until it reached 7%. After that, 7a and 7b lines reared on medium with 7% NaCl medium as described previously^[41,43,47]^. The fly lines were reared in cylindrical glass jars 0.25 L (0c, 2a-2c, and 4a-4c) or population cages 165 mm × 165 mm × 250 mm (0a-0b, 7a-7b) with medium and water.

In this way, the following lines of flies were tested: 3 control lines reared on a medium without salt (0a, 0b, and 0c), 3 lines reared on a medium with 2% NaCl (2a, 2b, 2c), 3 lines - with 4% NaCl (4a, 4b, 4c) and 2 lines - with 7% NaCl (7a and 7b). Two and 4% NaCl concentrations - were moderate, and 7% - was high. Additionally, five samples of substrates on which flies lived for 2 weeks were tested: s0b (substrate was processed by flies from 0b line), s0c, s2a, s4a, and s7a. Studied substrates consisted of flies’ medium and metabolites, on which their specific microbial community developed.

Thirty adult flies at the age of 7 days after the eclosion were taken from each fly line. This age of flies was chosen not by chance: previously, it was shown that flies emerged from the pupa being almost sterile^[2]^ and formed a microbiome typical for their line by the age of 7 days by eating previously processed substrate^[43]^. To remove the substrate particles and the microbial cells from the surface of the body, the flies were washed in 10 mL of sterile water on a MultiReax vortex (Heidolph, Germany) at 1,700 rpm for 15 min. After that, the flies were homogenized in 3 mL of sterile water and treated by the vortex. To study the microbial communities associated with the substrate, 30 mg of the substrate from the surface was resuspended in 3 mL of sterile water and homogenized in the same way as the fly samples. The taken mass of the substrate was approximately equivalent to the mass of 30 flies taken to prepare the homogenate.

### Isolation and identification of the dominant representatives of microbial groups

The total number of cultivated aerobic bacteria was determined by plating the fly homogenates using the dilution-to-extension method on Petri dishes with MRS agar medium of the following composition, g/L: peptone - 12.5; yeast extract - 7.5; glucose - 20.0; KH_2_PO_4_ - 2.0; dibasic ammonium citrate - 2.0; sodium acetate - 5.0; MgSO_4_ - 0.2; MnSO_4_ - 0.05; agar - 20.0; tap water, pH 7.0. Amphotericin B (1 mg/L) was added to the medium to inhibit yeast growth. The inoculated Petri dishes were incubated for 5-10 days at room temperature (20-25 °C), and after that the colonies were counted. Colony growth experiments were performed in 3 dilutions (3 replicates each) for each sample of flies or substrate.

Colonies obtained from the highest dilutions and containing cells of different morphotypes were used for the inoculation of a fresh MRS agar medium. DNA was isolated from the obtained pure cultures using the Fast DNA Spin Kit for Soil (MP Biomedicals, California, USA).

For amplification of the *16S rRNA* gene, biomass of pure bacterial culture grown for 2-3 days was subjected to boiling in Tris-EDTA buffer (pH 8.0) which contained 5% Triton X-100, and homogenization by mechanical destruction with sterile glass beads (250-300 μm in diameter) using a Homogenizer Minilys (Bertin Instruments, Montigny-le-Bretonneux, France) at 5,000 rpm for 30 s. The resulting homogenate was centrifuged, and the resulting supernatant was used as a DNA template for PCR^[48]^.

For amplification of the gene of interest, the primers 27f (5’-AGAGTTTGATCCTGGCTCAG-3’) and Un1492r (5’-ACGGYTACCTTGTTACGACTT-3’) were used^[49,50]^. PCR amplification program was 94 °C 3:00, 55 °C 0:30, 72 °C 1:00, 94 °C 0:30, for 37 cycles, following 55 °C 0:30, 72 °C 5:00.

The PCR products were purified and sequenced by the Research and Production Company “Evrogen” (Moscow, Russia) using the 1100 r^[51]^. The editing of the nucleotide sequences was carried out using Chromas Lite 2.01. The Clustal Omega and the BLAST algorithm from the GenBank database were used to align, compare, and identify nucleotide sequences. The obtained bacterial nucleotide sequences were deposited to NCBI GenBank under accession numbers OR272523-OR272526.

The yeast component of fly microbiota was described earlier in the paper by Dmitrieva *et al.*^[44]^.

### Bacterial diversity identification and estimation

Estimation of bacterial microbiota composition was performed by NGS sequencing of variable V4 region of *16S rRNA* gene. DNA isolation from flies and substrate homogenates was performed using the DNeasy PowerLyzer Microbial Kit (Qiagen, Hilden, Germany). Amplification of the V4 region of the *16S rRNA* gene was carried out using two primers consisting of the Illumina TruSeq sequencing primer adapters and 515F/Pro-mod-805R primer sequences: Forward 515F (5’-GTGBCAGCMGCCGGGTAA-3’)^[52]^ and Reverse Pro-mod-805R (5’-GACTACNVGGGTMTCTAATCC-3’)^[53]^. PCR amplification was performed as follows: 32 cycles of denaturation at 95 °C for 25 s; primer annealing at 56 °C for 20 s; DNA synthesis at 72 °C for 30 s, and a final elongation at 72 °C for 20 min^[54]^. High-throughput sequencing of the libraries was performed with MiSeq Reagent Micro Kit v2 (300-cycles) MS-103-1002 (Illumina, USA) on a MiSeq sequencer (Illumina, USA) according to the manufacturer’s instructions. The raw reads were processed as described by Gavrilov *et al.*^[55]^ and analyzed using the SILVAngs service with default parameters (SILVA138.1 SSU database, https://ngs.arb-silva.de/silvangs/). The obtained nucleotide sequences were deposited to NCBI BioProject under accession numbers PRJNA999597.

For each sample, profiling was made in two replicates, and the percentage of bacteria of each genus was averaged over them. *Wolbachia*, an intracellular symbiont of *D. melanogaster* (on average 15% in each sample), was excluded from the further analyses. Bacterial taxa whose percentage did not exceed 5% in any of the lines were assigned to the “Other” section.

### Assessment of the salt tolerance of the dominant bacterial groups

To assess salt tolerance, we grew the isolated strains in liquid MRS medium with 0%, 2%, 4%, and 7% NaCl stirring on a shaker (200 rpm); 4 replicates for each isolated strain on each salinity level. Strains’ growth was evaluated by measuring the optical density on a KFK-3-01 30M3 photoelectric colorimeter at a wavelength of 600 nm. Salt tolerance was evaluated as the ratio (in percentage) of the optical density of a culture grown on a medium with NaCl to the optical density of a culture grown on a medium without NaCl.

### Data analyses

The diversity of microbiota in each sample was assessed using the Shannon species diversity index^[56]^.

The similarity of flies- and substrate-associated microbial communities was assessed using the coefficient of biocenotic similarity (K)^[57]^, which considers both the abundance and species composition of microorganisms in the compared samples [Equation (1)].

**Figure:**
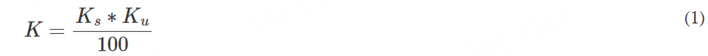


Where *K_s_* is the species composition similarity coefficient [Equation (2)], *K_u_* is the coefficient of relative species abundance similarity [Equation (3)].

**Figure:**
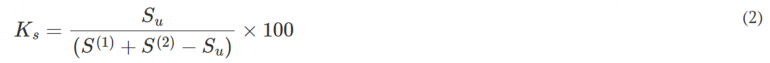


Where *S*^(1)^, *S*^(2)^ are the numbers of species in the first and second samples, respectively, *S_u_* is the number of species common for compared samples.

To calculate the coefficient of relative species abundance similarity, we calculate the percentage of each species of microorganisms in each sample, then select a smaller value for the species common to both samples and sum up the selected values:

**Figure:**
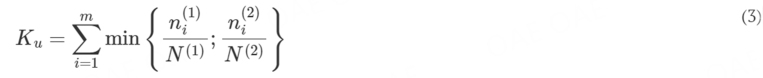


Where *m* is the number of common species in two samples, *N*^(1)^ and *N*^(2)^ are the total numbers of microorganisms in the first and second samples, respectively, *n_i_*^(1)^ and *n_i_*^(2)^ are the numbers of the *i*-th species of microorganism in the first and second samples, respectively.

The difference in salt tolerance was examined with an analysis of variance (ANOVA). Multiple comparisons were made with Tukey’s HSD test. To plot the figures, the values for the replicates of plating experiments were averaged. The possible interrelation between the bacterial and yeast components of the fly microbiome was checked by pairwise correlations between the following five variables: salt concentration, bacterial abundance, yeast abundance, bacterial diversity, and yeast diversity (10 pairwise comparisons). The Spearman and Kendall correlation coefficients were calculated because the relationships between the variables are rather nonlinear. The results obtained based on both coefficients were very similar; the Spearman coefficients are given below. The significance of the correlation coefficient was checked using *t*-test.

## RESULTS

Bacterial communities associated with *D. melanogaster* adapted to the substrate with different salinity were studied through growth experiments and the analysis of the variable V4 region of the *16S rRNA* gene. The samples represented the following study variants - flies reared on the substrate without NaCl (3 lines), with 2% and 4% NaCl (3 lines each), with 7% NaCl (2 lines), and samples of the substrate processed by flies from 5 lines. The results revealed that the quantitative and qualitative composition of communities varied among different lines, sometimes significantly, even within replicates (in fly lines reared on the substrate with the same salinity), but several general trends were observed.

### The diversity of bacteria in the fly lines adapted to the substrate with different salinity and in the corresponding substrates

**Figure 1:**
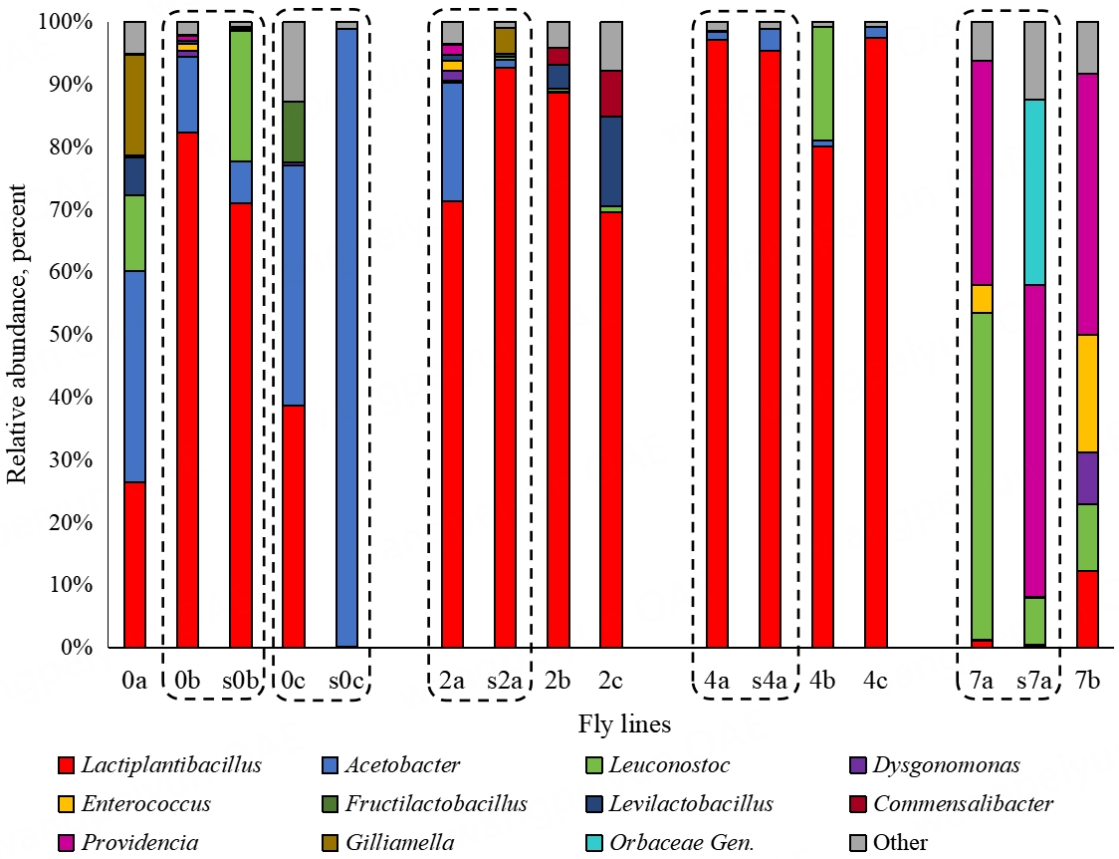
Composition of the bacterial microbiome in the fly lines adapted to the substrate with different salinity and in the corresponding substrates. 0a, 0b, and 0c - control lines reared on a medium without salt, 2a, 2b, 2c - lines reared on a medium with 2% NaCl, 4a, 4b, 4c - with 4% NaCl, 7a and 7b - with 7% NaCl. For each fly line, profiling was made in 2 replicates.

Bacterial communities associated with *D. melanogaster* differed significantly, in agreement with NaCl concentration in the substrate. Figure 1 represents the average relative abundance of bacteria of different taxa in the fly line denoted at the x-axis. Raw data are presented in Supplementary Table 1. In the absence of NaCl (lines 0a-0c), as well as at 2% and 4% NaCl concentrations (lines 2a-2c and 4a-4c), bacteria of *Acetobacter* and *Lactiplantibacillus* genera dominated in flies, and their relative abundance ranged from 69% to 99%. However, with the increase in NaCl concentration, the relative abundance of *Acetobacter* decreased, while that of *Lactiplantibacillus* grew, and at 4% NaCl, it became dominant. The composition of the fly microbiome changed entirely at 7% NaCl, being dominated by bacteria of *Leuconostoc* and *Providencia* genera.

The microbiomes of the lines reared under the same conditions were generally similar but differed in the relative abundance of *Lactiplantibacillus* and *Acetobacter*. The *Lactiplantibacillus* content varied from 26% to 82%, and *Acetobacter* - from 12% to 38% in control lines (0a-0c). Bacteria of *Gilliamella*, *Leuconostoc*, and *Levilactobacillus* genera (16%, 12%, and 6%, respectively) were detected in line 0a, and the representatives of the genus *Fructilactobacillus* (10%) were present in line 0c. Lines reared on the substrate with 2% NaCl contained 69% to 89% lactic acid bacteria, while *Acetobacter* was present only in line 2a (19%). *Acetobacter* was replaced by *Levilactobacillus* (4% and 14%) and *Commensalibacter* (3% and 7%) in lines 2c and 2b. In lines reared on the substrate with 4% NaCl, *Lactiplantibacillus* absolutely dominated, and *Leuconostoc* was found in significant amounts only in the microbiome of line 4b (18%). Thus, the main microorganisms associated with *D. melanogaster* were *Lactiplantibacillus* and *Acetobacter*, and the proportion of *Acetobacter* decreased to a few percent (in the case of 4% salt) with the increase in substrate salinity. Completely different results were observed in two lines reared on the substrate with 7% NaCl. *Acetobacter* was absent; *Lactiplantibacillus* accounted for only 1% to 12% of the total prokaryotic community. Instead, bacteria of the genera *Leuconostoc* (52% and 11%), *Providencia* (36% and 42%), *Enterococcus* (4% and 19%), and in one case, *Disgonomonas* (8%) dominated [Figure 1].

The bacterial composition of the substrate with different salinity partially corresponded to the composition of microbiomes of *D. melanogaster* reared on this substrate, but there were some differences [Figure 1]. In one of the samples from the control line (s0a), 21% of bacteria of the genus *Leuconostoc* were present and were absent in the microbiome of the corresponding fly line (0a). The s0b substrate was dominated by *Acetobacter*, while the microbiome associated with the flies (0b) contained much less *Acetobacter* and included mainly lactobacilli (*Lactiplantibacillus* and *Fructilactobacillus*). Substrate and fly microbiota composition were similar at 2 and 4% NaCl - *Lactiplantibacillus* dominated. The substrate with 7% NaCl contained the genera *Leuconostoc* and *Providencia*, which were also found in flies, but representatives of the *Orbaceae* family absent in fly microbial communities were detected in the substrate in significant amounts (almost 30%).

### Salt tolerance of bacterial strains associated with *D. melanogaster*

Three bacterial strains were isolated from the highest positive dilutions of the fly homogenate. Based on the analysis of full-length *16S rRNA* gene sequence, the strains were identified as the representatives of *Lactiplantibacillus plantarum*, *Leuconostoc pseudomesenteroides*, and *Acetobacter pasterianus*.

**Figure 2:**
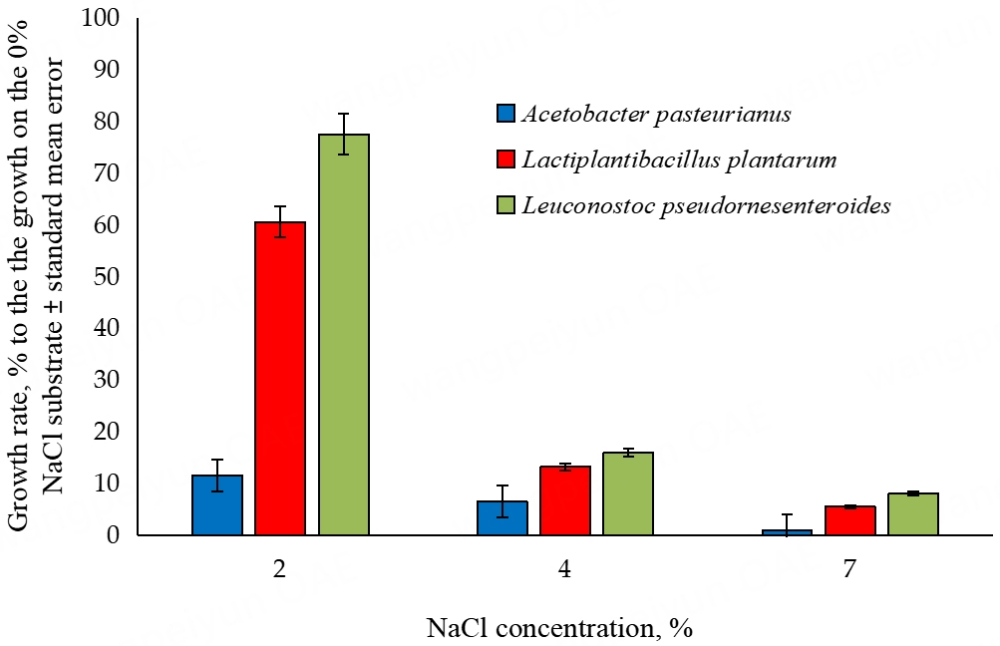
The effect of NaCl on the growth rates of dominant species of bacteria associated with flies. Bars represented the average optical density ratio of a culture grown on a medium with NaCl to that grown on a medium without NaCl (*n* = 4). The difference between all groups is statistically significant, *P* < 0.0005.

The salinity tolerance of these isolates was tested in four replicates for each isolated strain on each salinity level [Figure 2, Supplementary Table 2]. ANOVA showed that the salinity tolerance strongly depended on the isolate, salt concentration, and interaction of these factors (*P* < 2 × 10^-16^ in all three cases). It was found that *A. pasteurianus* was the most sensitive to salt (90% growth decrease at 2% NaCl). *L. plantarum* was more resistant to increased salinity, as at 2% NaCl, its growth decreased only by 40%. *L. pseudomesenteroides* was the most resistant to the salinity of the substrate compared with the other two strains. However, its growth also decreased significantly with the increase of the media salinity. According to Tukey’s HSD test, the difference between all groups is statistically significant, *P* < 0.0005.

### The total number of bacteria and yeasts in the fly lines adapted to the substrate with different salinity and in the corresponding substrates

The number of aerobic and aerotolerant bacteria in *D. melanogaster* homogenates of different lines determined by direct plating varied from 10^4^ to 10^7^ CFU per 1 fly. The bacterial count depended on the concentration of NaCl in the substrate, but it could differ by an order of magnitude in lines reared at the same salt concentration [Table 1, Supplementary Table 3]. In *D. melanogaster*, the number of bacteria decreased with the increase in NaCl concentration from 0% to 2%, and increased and exceeded the number of bacteria in the control lines with an increase in NaCl from 2% to 4%. However, at 7%, it decreased again to the level of control flies (0% NaCl). A different trend was observed in the substrate samples: the number of bacteria was the highest on the substrate without salt and the lowest at 7% NaCl. At 4% NaCl, the number of bacteria was higher than that at 2%, similar to the pattern observed in the flies.

**Table 1:** The abundance of bacteria and yeasts in the fly lines adapted to the substrate with different salinity and in the corresponding substrates

**NaCl concentration, %**	**Fly or substrate sample**	Total number of bacteria, CFU per fly or 1 mg of substrate ± standard deviation^*^	Total number of yeasts, CFU per fly, or 1 mg of substrate ± standard deviation^**^^,^^***^
0	0a	895,000 ± 161,142	3,480 ± 972
	0b	2,860,000 ± 245,085	560 ± 246
	0c	7,585,000 ± 2,741,660	0
	s0b	97,700,000 ± 8,808,140	20 ± 63
	s0c	13,021,000 ± 1,606,238	1,020 ± 670
2	2a	47,200 ± 11,013	96,740 ± 10,688
	2b	9,900 ± 2,094	191,160 ± 60,733
	2c	20,100 ± 2,051	295,300 ± 83,388
	s2a	151,400 ± 52,244	118,960 ± 20,188
4	4a	2,107,500 ± 229,982	133,400 ± 32,146
	4b	21,840,000 ± 2,633,755	139,120 ± 41,235
	4c	6,005,000 ± 599,138	504,440 ± 149,799
	s4a	4,492,500 ± 803,632	201,240 ± 59,948
7	7a	8,295,300 ± 957,967	52,306 ± 12,370
	7b	1,390,400 ± 422,926	2,068 ± 1,029
	s7a	3,100 ± 354	29,596 ± 11,460

^*^30 flies or 3 mL of the substrate, three dilutions, three replicates each; ^**^30 flies or 3 mL of the substrate, ten replicates; ^***^the data from the paper of Dmitrieva *et al.*^[44]^.

For comparison, the number of yeasts, both in *D. melanogaster* homogenates and on the substrate, increased with the rise in salinity to 2% and 4% but decreased at a salt concentration of 7% [Table 1, Supplementary Table 4]^[44]^.

In flies from lines 0a-0c, 4a-4c, and 7a-7b, the number of bacteria was 1-3 orders of magnitude higher than that of yeast. Conversely, in three lines 2a-2c, there were significantly more yeasts than bacteria (2-19 times).

### Quantification of similarities between flies and substrates microbial communities

We quantified the similarity of the microbial communities of flies and their substrates by the coefficient of biocenotic similarity [Table 2].

**Table 2:** The coefficient of biocenotic similarity for the flies and the corresponding substrate

**NaCl concentration, %**	**Fly and substrate samples**	**Coefficient of biocenotic similarity. Bacteria**	Coefficient of biocenotic similarity. Yeasts^*^
0	0b and s0b	0.48	0
	0c and s0c	0.26	0
2	2a and s2a	0.32	0.74
4	4a and s4a	0.98	0.33
7	7a and s7a	0.25	0.09

^*^Estimated based on data from the paper of Dmitrieva *et al.*^[44]^.

The bacterial communities of the fly line and corresponding substrate were most similar at a salinity of 4% (for s4a and 4a, the similarity score was 0.98), mainly due to the predominance of species of the genus *Lactiplantibacillus* in both samples. The lowest similarity (0.25) was observed at 7% salinity (s7a and 7a), probably reflecting the difference between the conditions on a high salty substrate and the fly body as habitats. An intermediate level of similarity was observed at salt concentrations of 0% (for 0b and s0b, 0c and s0, the similarity score was 0.48 and 0.26, respectively) and 2% (for s2a and 2a, the similarity score was 0.34). The similarity scores were different in the yeast part of the microbial community. The coefficients of similarity of the yeast community in control flies and their substrates were equal to zero. The similar index at 7% NaCl was 0.09 (very low). The similarity of the yeast community of the 4% salinity substrate and flies was low (0.33), while for the 2% salinity substrate and flies, it was the highest (0.74).

### The diversity of yeast and bacterial microbiota in the fly gut

**Figure 3:**
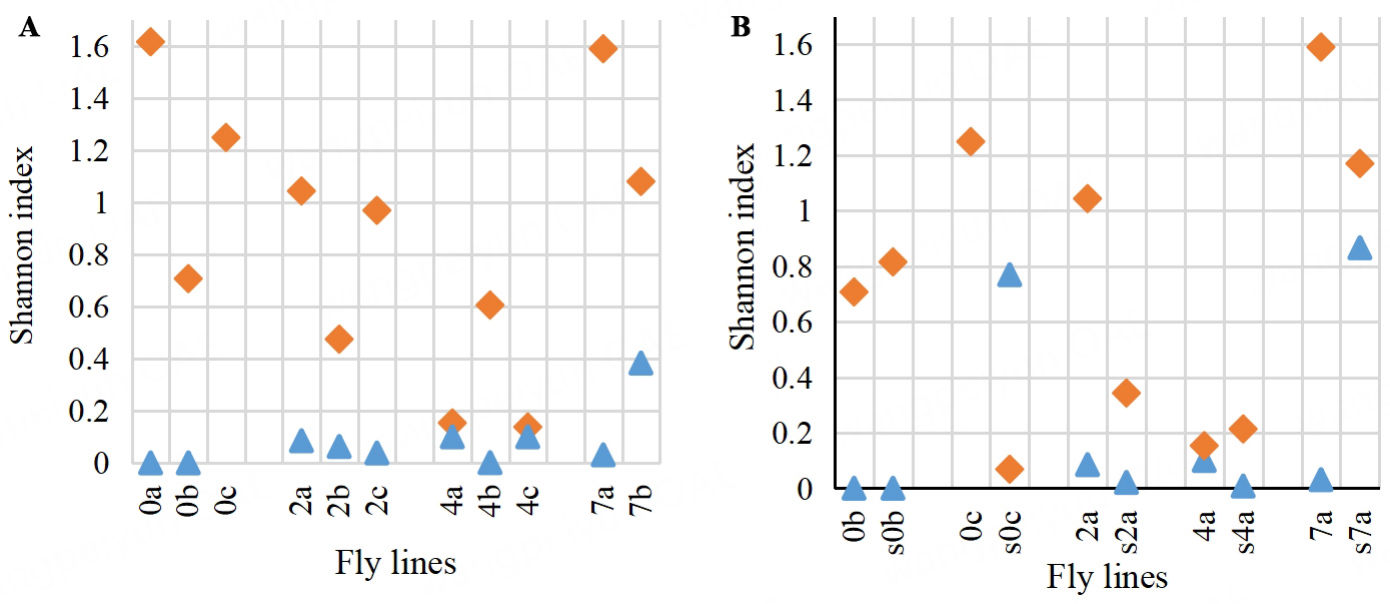
Diversity assessment (Shannon index) for the bacterial (diamonds) and yeast (triangles) components of microbial communities: (A) flies; (B) flies and corresponding substrates. Designation of the fly lines is the same as in Figure 1.

The diversity of bacterial and yeast microbiota was assessed by the Shannon index. The diversity of the bacterial community decreased on average with the increase of salinity from 0% to 4% and rose significantly with an increase in salt concentration to 7% [Figure 3A, diamonds]. The bacterial microbiota of flies was more diverse than the substrate microbiota for lines 0b and 4a, and the opposite was the case for the other fly lines studied [Figure 3B, diamonds].

The yeast communities of the flies, as well as the corresponding substrates, were less diverse than the bacterial ones [Figure 3, triangles], except for the s0c sample. The diversity of yeasts in the substrate with 2% and 4% NaCl was lower than in the corresponding flies. However, with an increase in salt concentration to 7%, the yeast community of the substrate became much more diverse than that of the flies.

### Relative microbial abundance in one fly to 1 mg of substrate

**Figure 4:**
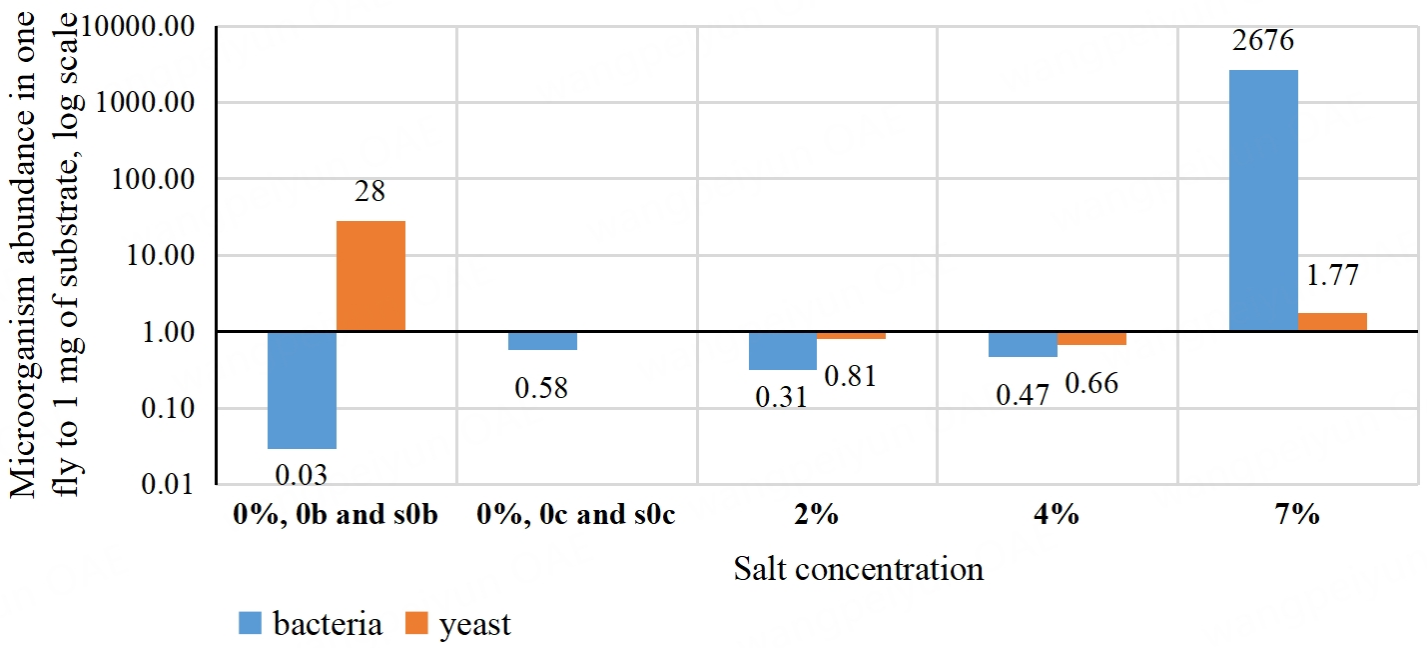
The ratio of the microorganism abundance in one fly to 1 mg of substrate. Sample sizes for studying bacteria were 30 flies or 3 mL of the substrate, 3 dilutions, 3 replicates each; for yeasts - 30 flies or 3 mL of the substrate, 10 replicates.

The relative bacterial and yeast growth efficiencies on the substrate with different salinity and in the corresponding flies were estimated [Figure 4]. The similar ratio for yeast did not increase with an increasing salt concentration in the substrate - at salt concentrations from 2% to 7%, one fly contained about the same amount of yeast as 1 mg of the substrate.

### Pairwise correlations between different microbial parameters

To assess the possible interrelation between the bacterial and yeast components of the fly microbiome for the corresponding 11 samples, we tested all possible pairwise correlations between the following five variables: salt concentration, bacterial abundance, yeast abundance, bacterial diversity, and yeast diversity (10 pairwise comparisons). At the 5% significance level, only the following two correlation coefficients were nonzero: salt concentration - yeast diversity (positive correlation) and yeast abundance - bacteria diversity (negative correlation). All other correlation coefficients were insignificant [Table 3].

**Table 3:** Pairwise Spearman correlations calculated for 11 fly samples

**Variables**	**Salt concentration**	**Bacterial abundance**	**Yeast abundance**	**Bacterial diversity**	**Yeast diversity**
Salt concentration	1				
Bacterial abundance	+0.30	1			
Yeast abundance	+0.33	-0.25	1		
Bacterial diversity	-0.20	+0.04	-0.66^*^	1	
Yeast diversity	+0.69^*^	-0.32	+0.47	-0.47	1

^*^5% significance level.

Considering that the microbiomes of the flies reared on the substrate with 7% NaCl concentration (7a-7b) were contrastingly different from all the others, the calculations were repeated for 9 samples, excluding these two. In this case, five pairwise correlations out of 10 possible were significant at the 5% significance level: salt concentration - yeast diversity (positive correlation) as in the case with 11 samples; salt concentration - yeast abundance (positive correlation), salt concentration - bacterial diversity (negative correlation), yeast abundance - yeast diversity (positive correlation), bacterial diversity - yeast diversity (negative correlation). The pair yeast abundance - bacteria diversity (negative correlation), which was significant at a 5% significance level on a set of 11 samples, became nonsignificant (significant only at a 10% significance level) on a set of 9 samples [Table 4].

**Table 4:** Pairwise Spearman correlations calculated for 9 samples of flies reared on the substrate with 0%, 2%, and 4% NaCl

**Variables**	**Salt concentration**	**Bacterial abundance**	**Yeast abundance**	**Bacterial diversity**	**Yeast diversity**
Salt concentration	1				
Bacterial abundance	+0.15	1			
Yeast abundance	+0.74^*^	-0.27	1		
Bacterial diversity	-0.79^*^	-0.10	-0.66^**^	1	
Yeast diversity	+0.80^*^	-0.29	+0.70^*^	-0.73^*^	1

^*^5% significance level; ^**^10% significance level.

## DISCUSSION

The microbiome of *D. melanogaster* is a classical subject of genetic and microbiological research. However, most studies of fly-associated microbes focus either on bacteria or on yeast, whereas attempts to study both components simultaneously are quite rare and vulnerable to criticism. For example, using the same selective medium for plating both groups of microorganisms has been a source of critique^[33]^. Our study confirmed the low diversity of fly-associated bacteria, predominantly represented by culturable and well-studied species. The bacterial microbiome of the control lines is dominated by *L. plantarum* and *A. pasteurianus*, the two most common species associated with laboratory-grown fruit flies. Other species include *L. pseudomesenteroides*, a species reported earlier from *D. melanogaster* microbiota, and *Giliamella* sp., unusual for fruit flies but known as symbionts of honeybees and bumblebees’ intestines^[58]^. *D. melanogaster*, like bees, are insects closely associated with sugar-rich plant substrates; therefore, their gut microbiome might be similar in some ways, especially on the control and low salinity substrate (2%), as observed in this study. Moreover, our results are compatible with the idea that the fly microbiome is not entirely transient, as its composition, in most cases, differs significantly between the flies and the substrate they inhabit.

We found that an increase in substrate salinity to 2%-4% results in a decline in diversity of the fly-associated bacterial microbiome [Figure 3A, diamonds], as well as in a decrease of *Acetobacter* to lactic acid bacteria ratio both in the flies and in the corresponding substrates. This may be partially caused by the high sensitivity of *A. pasteurianus* to salinity, as confirmed experimentally [Figure 2]. The results obtained on a substrate with extremely high salinity (7%) show significant changes in the fly-associated bacterial microbiome. The potentially beneficial symbionts, *Lactoplantibacillus* and *Acetobacter*, were replaced by other bacteria, including *Providencia*, which can be pathogenic for *D. melanogaster*^[59]^, and moderately salt-tolerant *Leuconostoc* and *Enterococcus* strains. Representatives of those two genera are typical symbionts of the human intestine and are also sometimes found in the intestines of wild fruit flies^[18]^.

**Figure 5:**
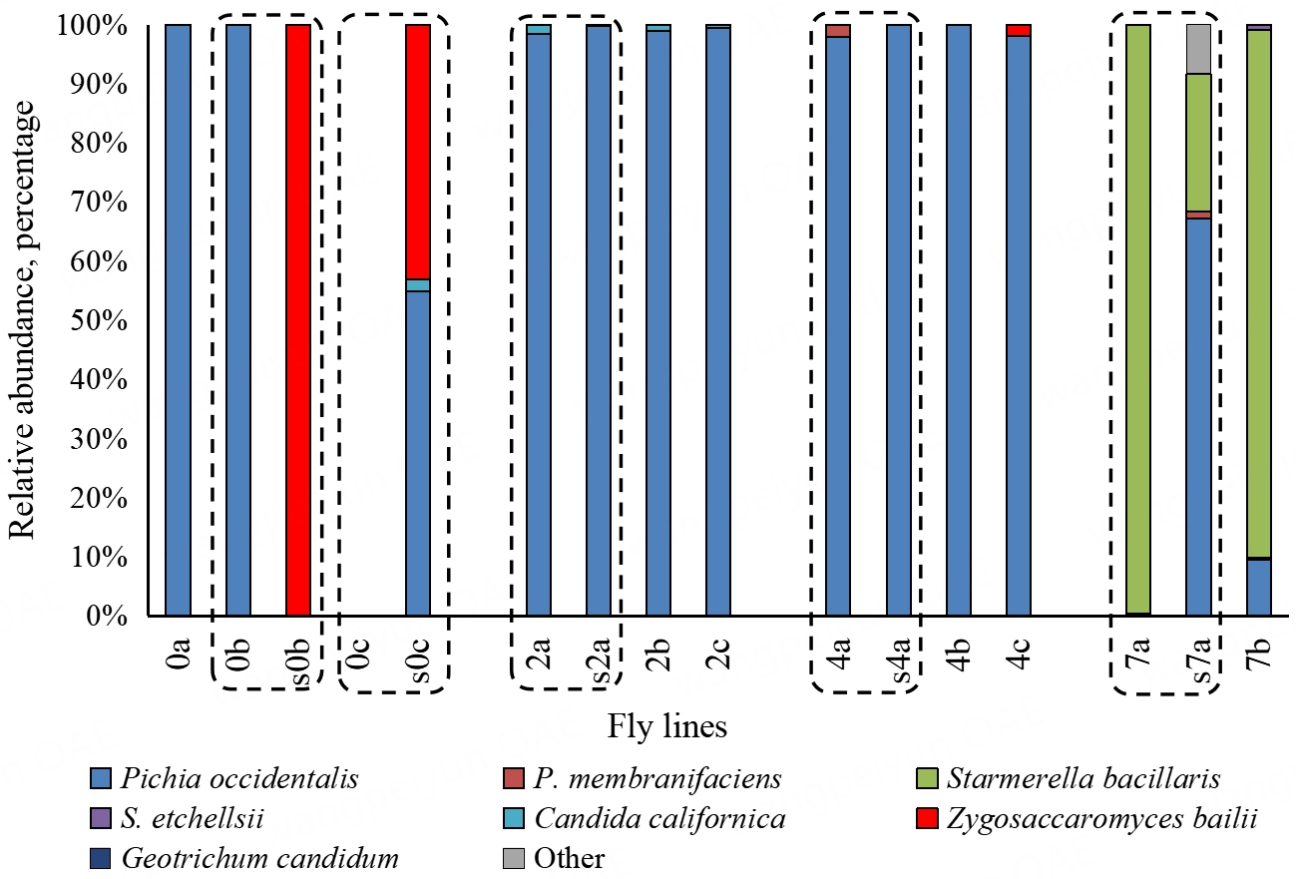
Composition of the yeast microbiome in the fly lines adapted to the substrate with different salinity and in the corresponding substrates (the data from the paper of Dmitrieva *et al.*^[44]^). 30 flies or 3 mL of the substrate, ten replicates. Designation of the fly lines is the same as in Figure 1.

The detailed description of the yeast component of the microbiome of the same *D. melanogaster* lines was published earlier^[44]^, allowing for a comparison of the two components [Figure 5]. Yeast populations of flies reared on low salinity substrates (0%-4%) consist almost entirely of one species, *Pichia occidentalis*, except for one fly line in which no yeast was detected (0c). However, at 2% and 4% salt concentrations, three other species (*Candida californica*, *Zygosaccharomyces bailii*, and *P. membranifaciens*) appeared as minor components. At the extremely high salt concentration (7%), *Starmerella bacillaris* became the dominant yeast species in flies, while the percentage of *P. occidentalis* drastically decreased. The minor components also changed - *C. californica* and *Z. bailii* were not detected, while *S. etchellsii* and *Geotrichum candidum* were observed.

The yeast populations of the substrate and flies differed significantly at the minimal (0%) and maximal (7%) salt concentrations but were similar at intermediate levels (2% and 4%) [Figure 5]. The substrate s0b was inhabited only by the yeasts *Z. bailii*, while only *P. occidentalis* was found in the flies that lived on this substrate (0b). No yeasts were found in the 0c fly line, but their substrate (s0c) was inhabited by *P. occidentalis*, *Z. bailii*, and *C. californica* (55%, 43%, and 2%, respectively). *P. occidentalis* (67%) dominated the substrate (s7a) at 7% NaCl, but this species was scarce in flies. *S. bacillaris* was predominant in fly line 7a but comprised only 23% of the yeast community in the substrate (s7a) [Supplementary Table 4].

Overall, the composition of the fly-associated yeast microbiome undergoes significant changes as the salt concentration in the substrate increases. The diversity and abundance of yeasts in flies tend to increase with the rising salt concentration [Figure 3A, triangles; Table 1]. This is in accordance with previous findings that certain yeast species may help *D. melanogaster* adapt to elevated salt concentrations^[41]^.

All identified yeast species are osmotolerant. The yeasts *C. californica*, *P. membranifaciens*, *P. occidentalis*, *S. bacillaris*, *S. etchellsii*, and *Z. bailii* can grow in a medium with a salt concentration of up to 10%^[60]^. The yeast-like fungus *Geotrichum candidum* is known to be sensitive to salinity. It can grow at a salt concentration of 7%, but the growth rate drops 14-fold compared to the salt-devoid medium^[61,62]^.

The substrate processed by flies for two weeks serves as a reservoir of microorganisms that enter the fly intestines and are subsequently excreted. Our comparison of bacterial diversity in substrate and flies did not reveal a general regularity [Figure 3B, diamonds]. In line 0b, the diversity of bacteria in the substrate was slightly higher than in the flies (due to the presence of *Leuconostoc*). In the other control line, 0c, the bacterial substrate community was almost a monoculture (99% *Acetobacter*), possibly because the yeast community was one of the most diverse [Figure 3]. At the same time, the bacterial community of the corresponding flies was quite diverse (perhaps due to the absence of yeast). The diversity of the bacterial communities in the substrates with 2% and 7% NaCl was lower than that of the corresponding flies, whereas, at a salt concentration of 4%, the situation was the opposite.

In all samples except for s0c, the diversity of the yeast microbiota was lower than that of the bacteria. Just as for bacteria, no clear relationship between the yeast diversity of flies and substrates was found. The correlation analysis [Tables 3 and 4] revealed the possible antagonism between the bacterial and yeast components of the fly microbiome. In samples with a relatively high abundance and/or diversity of yeasts, the bacteria tended to be less diverse, and vice versa. The bacterial component of the microbiome appears to be more sensitive to the salt concentration than the yeast component.

As the salinity of the substrate increases, the abundance of bacteria tends to become higher in the flies than in the substrate [Figure 4, blue bars]. Apparently, the saltier the substrate, the smaller the contribution of bacteria entering the intestine with the substrate to their total number in the fly. The number of bacteria per 1 mg of substrate was three orders of magnitude less than the number of bacteria per fly in the sample with 7% NaCl. A fruit fly weighs just about 1 mg, and the weight of the intestinal contents is presumably much less; therefore, the ratio between bacterial abundance in the fly gut and the substrate is even higher. This may be due to the water the fly drinks, thus decreasing the salinity of the intestinal content compared to the substrate’s salinity. This probably makes it possible for less salt-tolerant bacteria to grow in the gut. In contrast, the relative abundance of yeast in the flies, compared to that in the substrate, did not change significantly with the increase in salt concentration [Figure 4, orange bars]. This is probably because yeasts are generally more salt-tolerant than bacteria, and flies may selectively consume yeast biomass from the substrate.

Our results suggest that as the salinity of the substrate increases, yeasts tend to play a more significant role in *D. melanogaster* adaptation to the environment, while the importance of the bacteria decreases. This aligns with the observation that, with the increase in salt concentration, the percentage of *Acetobacter* (which can be beneficial for flies) decreased, while the proportion of lactic acid bacteria (also potentially helpful) first increased (from 0% to 4% NaCl), and then drastically decreased at 7% NaCl. It is also in line with the presence of *Starmerella* yeasts in the fly microbiome at high NaCl concentrations, previously shown to help flies survive and reproduce on the salty substrate^[42,44]^. These yeasts may also assist the flies in resisting potentially pathogenic bacteria, such as *Leuconostoc* and *Providencia*, whose percentage increased at higher salinity.

The radical rearrangement of the fly microbiome with the increase in NaCl concentration from 4% to 7% (some correlations that are significant for 0% to 4% NaCl concentrations become insignificant when samples 7a-7b are included in the analysis) is apparently due to the fact that extremely high salt concentration drastically limits the growth of some groups of microorganisms, giving an advantage to other, more salt-tolerant groups [Table 3]. Most notably, the yeast found in this study is generally more tolerant to high salinity than most bacteria typical for the *D. melanogaster* gut.

The absence of *S. bacillaris* in lines 4a-4c and the presence of this yeast in lines 7a-7b are noteworthy. In 2017-2018, *S. bacillaris* dominated in lines 4a-4c^[41,42]^, but later, in 2019-2020 and the current study, it was no longer found in them. This change likely represents coevolutionary dynamics or different stages of adaptation of the holobiont (the insect host and its associated microorganisms) to the salty substrate. It can be hypothesized that the yeast *S. bacillaris* tends to develop in fly lines that have recently been switched to a saltier substrate (like lines 7a-7b) and have not yet adapted genetically. The yeasts may help the flies tolerate high NaCl concentrations at this early adaptation stage, as shown in our previous studies^[42]^. However, later, these beneficial symbionts may be lost by flies^[43]^ as the insects gradually develop genetic adaptations to the salty substrate. Further experiments are needed to test this hypothesis.

### Conclusions

In summary, we observed systematic changes in the composition and structure of the bacterial and yeast components of the *D. melanogaster* microbiome during the holobiont adaptation to the substrate with increasing salinity. These results can be attributed to the higher halotolerance of yeast compared to bacteria, leading to several changes: a gradual decrease in the proportion of beneficial bacteria and the emergence of potentially pathogenic ones, as well as the restructuring of the yeast community. Notably, the appearance of *S. bacillaris*, a yeast species, played a key role in the fruit fly’s successful adaptation to the 7% NaCl substrate. As the salt concentration increased, the yeast seemed to replace major bacterial species, providing the flies with beneficial metabolites and protecting them from potential pathogens.

### Study limitations

The empirical results reported herein should be considered in the light of some limitations. As was mentioned in the Materials and Methods section, the fly lines 0c, 2a-2c, and 4a-4c were reared in cylindrical glass jars 0.25 L, and the lines 0a-0b, 7a-7b in population cages 165 mm × 165 mm × 250 mm. On the one hand, undoubtedly, adaptation is a multi-factorial process, so a uniform setup should be kept in such studies. On the other hand, all other experimental conditions, except the rearing in a cage or jar, were the same, and no fluctuations were found in the data of the studied microbiota, which could be explained by rearing flies in a jar but not in a cage, and vice versa.

## DECLARATIONS

### Availability of data and materials

Supplementary Table 1: Bacterial microbiota composition by NGS sequencing of regions of *16S rRNA* genes; Supplementary Table 2: Salt tolerance of bacterial strains; Supplementary Table 3: The total number of culturable bacteria, plating, raw data; Supplementary Table 4: The total number of yeast species, plating, raw data.

### Copyright

© The Author(s) 2024.
